# Photodegradation of mixed organic dyes using Nb and W co-doped spray coated transparent conducting SnO_2_ thin films†

**DOI:** 10.1039/d5na00268k

**Published:** 2025-06-03

**Authors:** Gouranga Maharana, Yuvashree Jayavelu, Manavalan Kovendhan, D. Paul Joseph

**Affiliations:** a Department of Physics, National Institute of Technology Warangal Telangana State 506004 India paul@nitw.ac.in; b Department of Physics and Nanotechnology, SRM Institute of Science and Technology Kattankulathur Tamilnadu 603203 India

## Abstract

The implications of niobium (Nb) and tungsten (W) co-dopants on the physicochemical characteristics of spray deposited tin oxide (SnO_2_) thin films are investigated in order to evaluate it as an alternative transparent conducting oxide electrode. Co-doping with two cationic elements enables tweaking of the morphological and optoelectronic properties of SnO_2_ in thin film form. Spray pyrolysis is utilized for obtaining Nb (1 and 2 wt%) and W (fixed 4 wt%) co-doped SnO_2_ thin films on glass substrates. The surface wettability test shows that the cationic co-dopants W and Nb have influenced the SnO_2_ thin film by altering the surface features thereby reducing the contact angle value. The transmittance spectra indicate that pure SnO_2_ has a higher transmittance value of 83%, which diminishes upon addition of the W dopant and increases after Nb is introduced as a co-dopant. The linear four-probe measurement revealed that the films have a uniform sheet resistance around the centre with a low sheet resistance of ∼62 Ω □^−1^. The stabilized transparent conducting electrode's figures of merit of the deposited W and Nb co-doped SnO_2_ thin films are calculated and the promising results of photocatalytic tests for mixed dyes are discussed in correlation with the physicochemical and optoelectronic properties.

## Abstract 2

A potential strategy is devised to promote defect-mediated photocatalysis using LED light and immobilised spray-coated Nb and W codoped SnO_2_ thin-film photoelectrodes to degrade mixed dyes sustainably and with significant cycling stability.

## Graphical abstract

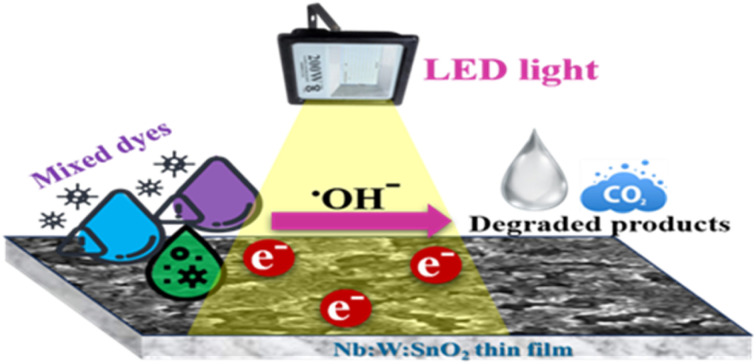

## Introduction

Advanced Oxidation Processes (AOPs) are highly efficient methods for disintegrating toxic dye effluents of textile industries. One AOP that has received quite a lot of attention is photocatalysis, which utilizes light energy to completely mineralize organic pollutants, making wastewater treatments more environmentally friendly and sustainable.^1,2^ Presently, several materials in their nanodimensions and morphology are explored intensely for photocatalysis.^3,4^ Based on the phase of the catalyst and catalyst type, photocatalysis is primarily divided into three different categories: homogeneous, heterogeneous, and enzyme/biocatalysis.^5,6^ Among these, the heterogeneous catalytic process holds significant industrial and environmental value as it is utilized in chemical transformations that are physiologically significant and for environmental remediation purposes such as pesticide removal, heavy element removal, and photocatalytic dye degradation.^7–9^ In general, solid substances that speed up a chemical reaction and do not alter once the reaction is finished are known as heterogeneous catalysts.

Scientific study into developing nanostructured oxides that increase the surface-to-volume ratio and hence promote interaction of photocatalysts with the organic pollutants has been inspired by the requirement to improve photocatalytic efficiency.^10^ In this aspect, thin films that are strongly adhered onto a substrate are developed, promoting the adsorption of organic pollutants over the surface of the photocatalyst, while improving light absorption and preventing recovery problems that are common to particulates. These thin films are coated over a substrate using a variety of methods, including chemical and physical deposition techniques. The spray pyrolysis method has various advantages, including simplicity, high film growth rates, minimal setup costs, mass production capability, and reproducibility.^11^ In this case, the precursor solution is sprayed over a heated substrate to form a thin layer of metal oxides. The high temperature during deposition induces oxide crystallization, thereby enhancing the transfer of the photoinduced charge carriers.^12,13^

There have been few investigations on the deterioration of mixed dye systems; nonetheless, when developing photocatalytic devices, dye quantification in such mixed systems and interactions with multiple dye counterparts must be considered. In a multi-dye system, interactions with other dyes can have a big influence on the photocatalytic processes. The utilization of Beer–Lambert's law for the dye's core absorption profile is problematic owing to its low accuracy in assessing the dye concentration in the mixture, rendering such tasks hard due to strong overlapping of spectra.^14^ A precise assessment and comprehension of dye concentrations are essential for maximizing the photocatalytic efficiency and guaranteeing efficient procedures.^15^

Recently, there has been a lot of interest in the production of nanocrystalline semiconductors due to their unique optical and spectroscopic properties.^16^ A semiconducting metal oxide with exceptional thermal, structural, and chemical stability is tin dioxide (SnO_2_). Pure SnO_2_ has a poor intrinsic conductivity due to its broad bandgap, which limits its effectiveness in applications that require strong electrical conductivity. The band gap of tin oxide can be modified easily by appropriate doping and by making them into nanostructured forms such as thin films, nanowires, and nanoparticles. Doping SnO_2_ with proper components is considered to be the most successful method among them, resulting in the formation of multiple valencies and oxygen vacancies and a change in optical response.^17^ The introduction of W ions substituting Sn ions within the SnO_2_ lattice increases free charge carriers, facilitates effective charge transport, and promotes enhanced electrical conductivity. The introduction of Nb can modify the optical characteristics of SnO_2_ (apart from improving the carrier concentration), potentially leading to better light absorption. In this study, Nb and W co-doped SnO_2_ thin films are deposited by the spray coating method and the structural, optical, and electrical transport aspects of the resultant films are explored by changing the Nb dopant concentration (1 wt% to 2 wt%) while retaining the W (4 wt%) concentration as a constant. Co-doping has changed the material's optoelectronic characteristics, enabling it to be utilized in the low-energy visible light energy domain. The performance of the stabilized films as a suitable photocatalyst for degrading multiple organic dyes is assessed in comparison to the photolysis process.

## Materials and methods

### Experimental

An automated spray pyrolysis unit with a static single nozzle was employed to deposit the pure and Nb:W co-doped SnO_2_ coatings in this work. The spray solution was made by dissolving 0.25 M stannous chloride dihydrate (SnCl_2_⋅2H_2_O) in ethylene glycol solvent. Tungsten chloride (WCl_6_) was chosen as the dopant source for W doping by dissolving it in distilled water and ammonia solution in a 3 : 1 ratio. Furthermore, niobium chloride (NbCl_3_) was used as a co-doping precursor to coat the co-doped SnO_2_ films. In order to deposit Nb:W co-doped SnO_2_ thin films, the W doping is fixed at 4 wt% and Nb was varied with 1 and 2 wt% by dissolving it in iso-propyl alcohol. To achieve a homogeneous and transparent solution, few HCl drops were added into all the precursor solutions. The final solution was loaded in the spray pyrolysis unit. Dust, residues and oil or grease that were adhered to the surface of the glass slides were removed by ultrasonically cleaning with mild detergent, deionized water, acetone, isopropyl alcohol, and ethanol in sequence for 10 minutes each. After cleaning, compressed air that had been moisture-filtered was used to blow dry the glass substrates. A high energy plasma cleaner (Model PDC-002, Harrick Plasma) has been utilized to eliminate the charged ionic impurities present on the glass substrate. The substrate was finally fixed in the coating chamber following the cleaning procedure and maintained at a substrate temperature of 380 ± 5 °C. A peristaltic pump was used to pump the precursor mixture solution to the spray nozzle with an inner diameter of 0.3 mm. The solution was sprayed over the hot glass substrate under optimal spray settings, such as a 30 cm spray nozzle to substrate distance and a pressure of 30 kg cm^−2^. About 600 sprays were performed for a spray duration of 1 s and at an interval of 30 s. Table 1 lists the codopant ratio along with the spray deposited film's sample ID.

**Table 1:** Nomenclature and the dopant ratio of the coated pure, W, and Nb:W co-doped SnO_2_ films

Sample ID	SnO_2_ (wt%)	W (wt%)	Nb (wt%)	At.% of elements from EDX
TO	100%	—	—	O = 69.2 and Sn = 26.3
WTO	96%	4%	—	O = 68.1, Sn = 29.2, and W = 2.5
NWTO1	95%	4%	1%	O = 68.3, Sn = 29.1, W = 2.8, and Nb = 0.01
NWTO2	94%	4%	2%	O = 68.4, Sn = 28.8, W = 2.6, and Nb = 0.09

### Characterization methods

An X-ray Rigaku SmartLab diffractometer with Cu K_α_ radiation of *λ* = 1.5406 Å was used to examine the crystallinity and crystal structure characteristics of all the coated Nb:W co-doped SnO_2_ films. A field emission scanning electron microscope (FESEM) with an energy-dispersive X-ray feature (EDX) (JEOL, JSM-IT800) and an optical profiler in non-contact mode (NanoMap 1000WLI) were used to explore the surface topology of the films. A Kratos/Shizmadu Amicus model ESCA 3400 validated the binding energy and oxidation states. A stylus profilometer was used to measure the thickness (Model: DektakXT, Bruker). A drop shape analyzer DSA25 was employed to analyze the contact angle (HOLOMARC, HO-IAD-CAM-01, India). A UV-Visible spectrometer (AnalytikJena, SPECORD 210 PLUS) was used to investigate the optical characteristics. Photoluminescence spectra (PL) of the samples were recorded under ambient conditions by using a Horiba Jobin Yvon Fluorolog-3-21 fluorimeter associated with an Xe arc lamp as an exciting source. The film's electrical transport features were obtained using a linear four probe (model: FP-01, SES Instruments Pvt. Ltd. India) and Hall effect (Ecopia HMS-3000) measurements.

### Photocatalytic setup

Photocatalytic tests were conducted in a rudimentary setup employing a cardboard enclosure and a visible white LED light (200 W) to assess the catalytic activity of the deposited thin film electrodes. Photocatalysis was carried out using an optimum film covering an area of 4 cm^2^ and a combination of dyes (Methyl Violet: MV, Malachite Green: MG, and Methylene Blue: MB) chosen as the model mixed dye pollutant. The mixture of dyes was made with a specific concentration (5 mg l^−1^) and volume (10 ml) of each dye and 6 ml of the mixed dye (MD) was collected for the test. The mixed dye solution's pH at room temperature was found to be 6.07 (slightly acidic). The film dipped in 25 ml of MD solution was directly exposed to visible light from an LED source (spectrum of the light source given in Fig. S1†) placed at a distance of 12 cm from the sample. The degrading MD solution concentration was measured through assessing the reduction in absorbance measured at an appropriate *λ*_max_ following each 30-minute illumination period using a UV-VIS spectrometer.

## Results and discussion

### Structural studies

**Fig. 1:**
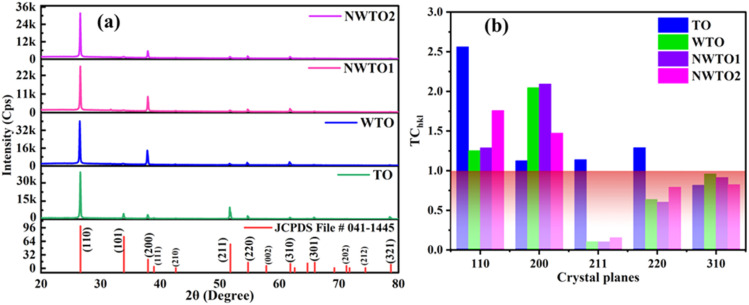
(a) XRD plots compared with the standard JCPDS file, and (b) texture co-efficient values of spray pyrolyzed Nb and W co-doped SnO_2_ films.

The spray-pyrolyzed pure and Nb:W co-doped SnO_2_ film's X-ray diffraction (XRD) reflections are shown in Fig. 1, which indicates granular nature with a tetragonal crystal structure (JCPDS file # 041-1445). Both the cationic dopants Nb and W substitute Sn^4+^ (69 pm) by ionizing into Nb^5+^ (69 pm) and W^6+^ (74 pm). The missing XRD peaks of additional phases such as Sn_2_O_3_ and SnO confirm that the doped films feature a pure tetragonal SnO_2_ phase. Impurity phases such as WO_3_ and Nb_2_O_5_ were not found in the patterns. Consequently, the Nb and W dopant ions have a fair chance of substituting the Sn sites of the SnO_2_ matrix. Regarded as the thermodynamically stable plane of the SnO_2_ crystal, the diffraction from the (110) plane has the highest intensity when compared to the other planes of all the other samples coated in this study.^18^ The dopant ion replacement at the host sites impacts the orientation of the crystal planes, and the addition of dopants has increased the (200) peak intensity while suppressing the (211) peak intensity. Our earlier research has demonstrated the suppression of the (211) plane caused by the addition of the W dopant into the SnO_2_ lattice.^19^ The relaxation of strain throughout the growth phase resulting from the inclusion of Nb into SnO_2_ may be the cause for the increase in (200) peak intensity.^20^ In order to analyse the preferential growth of granular thin films, the texture coefficient TC_*hkl*_ for the diffraction peaks is calculated based on Eq. 1,^21^ which is obtained from the diffraction intensities of every individual XRD pattern (Fig. 1b).1
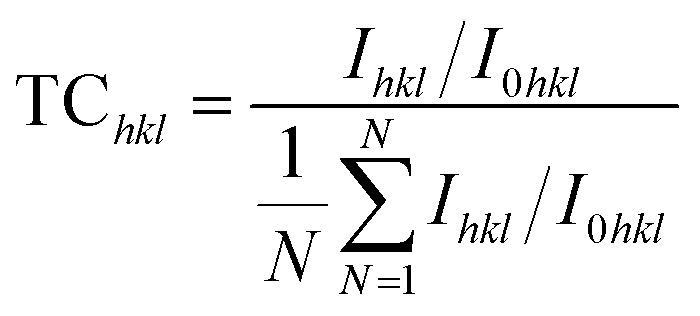
where *I*_*hkl*_ is the observed X-ray reflection intensity, *N* is the total number of reflections recorded in the corresponding XRD plot, and *I*_0*hkl*_ is the standard intensity reported in the JCPDS data file # 41-1445. A variance in the TC_*hkl*_ value greater than the unit value indicates growth that is preferable towards a particular orientation. The diffraction peaks (110) and (200) exceed the unit value of TC_*hkl*_ indicating a high degree of texture for all the deposited films. The reorientation of the crystallites caused by the insertion of Nb and W ions into SnO_2_ results in diminishing overall growth of every plane excluding the (200) plane for the co-doped samples. Eqn (2) is used for estimating the lattice parameters and the values are given in Table 2.2
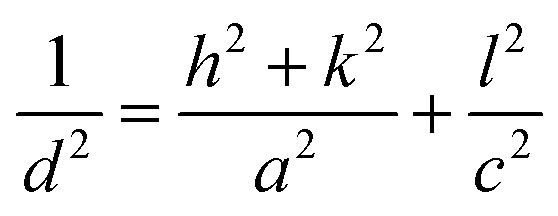
where *a* and *c* are the estimated lattice parameters, *h*, *k*, and *l* are the Miller indices, and *d* is the interplanar spacing. Table 2 lists the average crystallite size values estimated from the FWHM of all the peaks based on the Debye–Scherrer relation (eqn (3)).^22^3
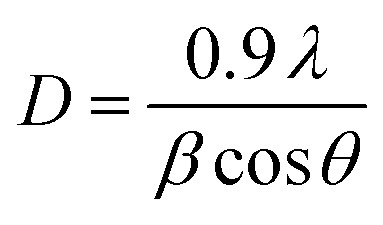
When the X-ray wavelength is *λ* (=1.5406 Å), the Bragg angle is *θ*, and the full width at half maximum is *β*.

**Table 2:** Structural features of pure and Nb:W co-doped SnO_2_ films along with their thickness and surface roughness values

Sample	Crystallite size *D* (nm)	Interplanar distance *d* (Å)	Lattice constants (Å)	Thickness (nm)	Surface roughness *r*_w_ (nm)
TO	37.85	3.347	*a* = *b* = 4.734, *c* = 3.205	884.4	270
WTO	35.60	3.358	*a* = *b* = 4.748, *c* = 3.192	516.8	217
NWTO1	35.56	3.347	*a* = *b* = 4.734, *c* = 3.196	516.7	207
NWTO2	36.36	3.350	*a* = *b* = 4.737, *c* = 3.183	497.6	178

The insertion of Nb and W dopants at Sn sites has led to the reduction of crystallite size from pure to co-doped SnO_2_ films. Dopants induce localized strain in the lattice, which may promote the development of smaller sized crystallites by increasing nucleation sites and decreasing the mobility of atoms in the vicinity.^23^

### Surface morphology

**Fig. 2:**
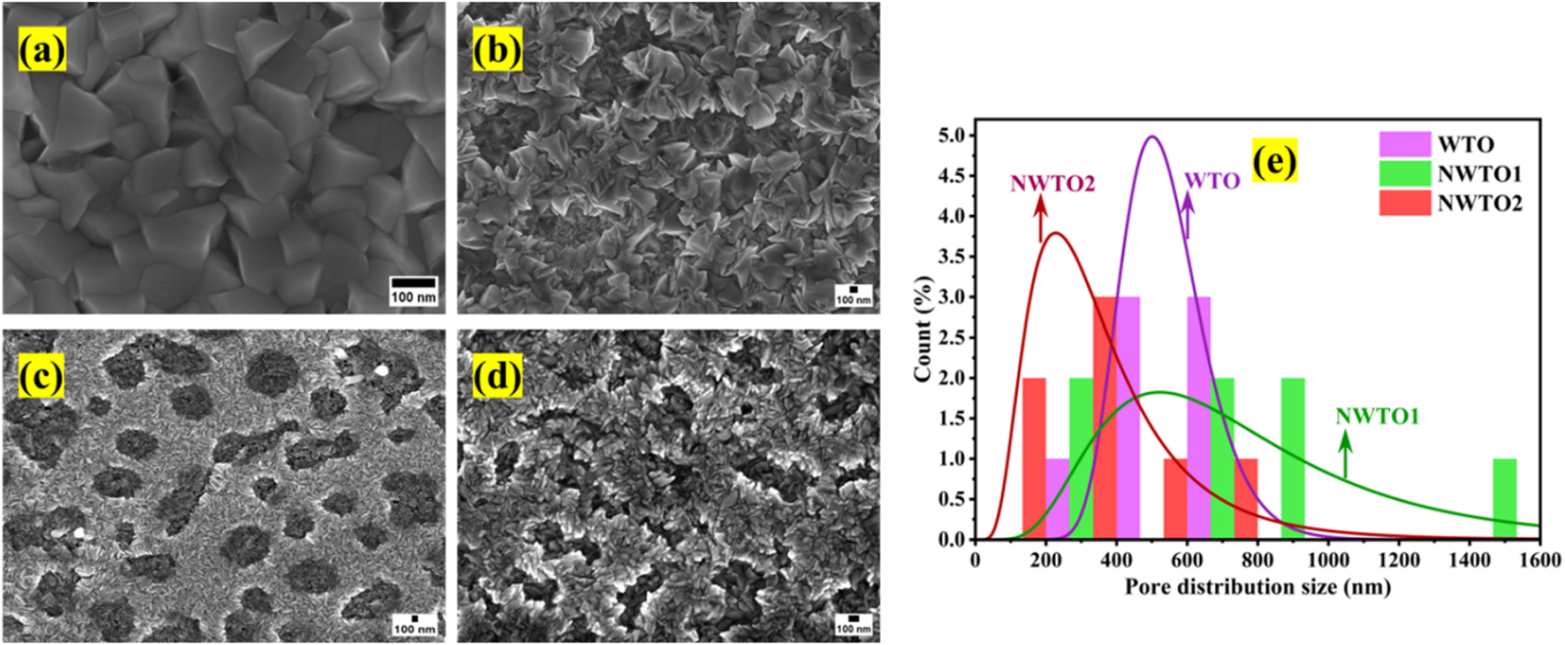
Field emission scanning electron micrographs of the spray-pyrolyzed pure and Nb:W co-doped SnO_2_ films (a–d) indicating variation in the surface features and pore size distribution plot for the doped films (e).

The FESEM micrographs of the pure and Nb:W co-doped SnO_2_ films with significant surface feature alterations following the changes in the dopant concentration are shown in Fig. 2a–d. Distribution of homogeneous particles of tetragonal shape on the surface of the pure SnO_2_ film indicates that the film is considerably denser. In contrast to the pure SnO_2_ film, the W doped SnO_2_ film's surface morphology tends to develop a network like structure. The multiple nucleations triggered by dopant insertion is a plausible means for the morphological transformation. This is consistent with the reduced crystallite size as observed in the XRD analysis due to dopant insertion. The shifting morphologies of the films validate it strongly that it is reliant on dopant choice of W, as stated in our earlier work.^19^ Each W-doped SnO_2_ film exhibits a distinct shape resembling a network like structure with a notable porosity. The pore size variation of all the doped films is shown in Fig. 2e, and the average pore size declines with increasing Nb co-doping content. The introduction of point defects, such as interstitials and oxygen vacancies, into the crystal lattice caused by doping SnO_2_ with different metal cations can alter the structure of the lattice locally, causing more strain and deformation that impedes the growth of bigger crystallites. Consequently, throughout the synthesis processes, the existence of these localized defects facilitates the development of smaller particle sizes.^24^ In addition, it is established by the combined assessment of XRD and FESEM that doping enables faster nucleation as indicated by the reduction in crystallite size, leading to multiple nucleation and smaller sized particle formation. Random grain orientation on the film's surface induces increased scattering, which could lower the optical transmittance. The elemental composition of the deposited films was assessed by EDX measurement that revealed the presence of Sn, W, and Nb elements in an appropriate ratio, as shown in Table 1.

### Surface topography

**Fig. 3:**
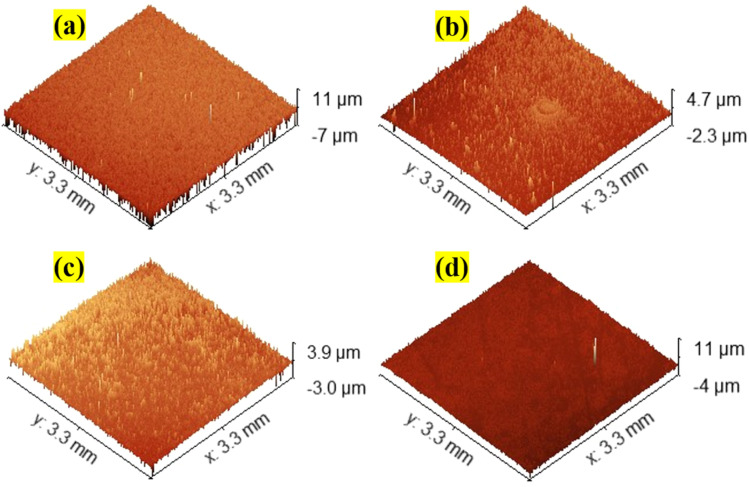
The 3D surface topography images assessed using an optical profiler for (a) TO, (b) WTO, (c) NWTO1, and (d) NWTO2 spray-coated films.

The optical profilometer's non-contact mode of operation uses a light source to analyse the surface features of the spray coated films. The SnO_2_ film's surface features have been described for a relatively wider region of 1 mm^2^ using this optical profiler. Fig. 3a–d display the 3D pictures of the surface morphology of all the coated pure and Nb:W co-doped SnO_2_ thin films. Gwyddion software (Version 2.61) is employed to compute the film's RMS surface roughness values^25^ (Table 2). Based on the test findings, it is observed that the inclusion of Nb and W as dopants reduces the surface roughness as compared to the pure SnO_2_ film. Thus, as observed in FESEM images (Fig. 2), the addition of W has resulted in multiple nucleations, causing a major variance in surface roughness owing to the nucleation and growth phenomena of the film. Rapid nucleation leads to thinner films because the particles tend to distribute more evenly across the substrate, in contrast to slower processes that result in thicker films as larger grains coalesce.^26^ This corresponds with the smaller particle formation (as observed in the FESEM image) and a drop in crystallite size as witnessed in the XRD data. This homogeneous distribution may inhibit excessive growth and retain a thinner film profile for the Nb:W co-doped SnO_2_ films. Nb and W doping has inherently modified the growth kinetics resulting in a relatively smoother surface with less variability in height across the film. This can be observed in the measurements of roughness and thickness, which often display comparable patterns due to the same origin.^26^ The thickness values of the coated films, measured with a stylus profilometer, are listed in Table 2.

### Surface wettability

Measuring the contact angle yields information about the surface wettability of the deposited pure and co-doped SnO_2_ films. A key determinant of the thin film's wettability is the angle of contact at the interfaces of fluid, vapor, and solid. Young established a relationship (eqn (4)) that offers essential details about the angle of contact at the interface under study.

**Fig. 4:**
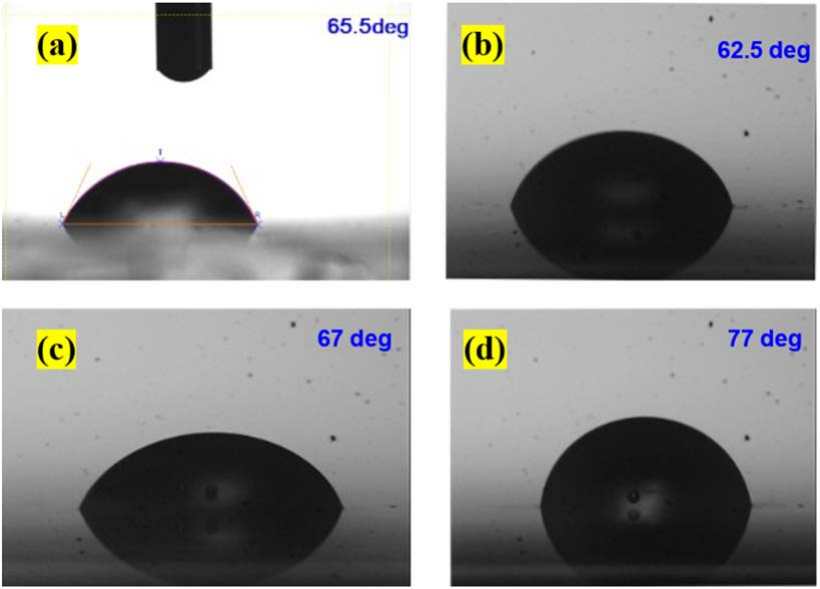
Surface wettability test by contact angle measurements for (a) TO, (b) WTO, (c) NWTO1, and (d) NWTO2 spray deposited thin films.

4 *Y*_sv_ = *Y*_sl_ + *Y*_lv_ cos *θ*

where *Y*_sl_, *Y*_sv_, and *Y*_lv_ are the interfacial energy per unit area of the solid–liquid interface, the solid–vapour interface, and the liquid–vapour interface, respectively, and *θ* is the angle of contact of the sample that is being investigated.^27^ Deionized water is used as the probe liquid to determine the angle of contact for a droplet of water on the surface of the pure and Nb:W co-doped SnO_2_ films (Fig. 4). These observations indicate that all of the films have a hydrophilic character and doping with W decreases the contact angle, whereas co-doping with Nb increases it afterward. The alteration in the contact angle of the deposited films is strongly dependent on the surface roughness.^28,29^ The equilibrium between the solid, liquid, and vapor phases at the interface is altered by surface roughness, which has an impact on the contact angle. The higher contact angle of TO as compared to the WTO film can be attributed to the high surface energy between the solid and liquid interface.^28^

### Optical properties

**Fig. 5:**
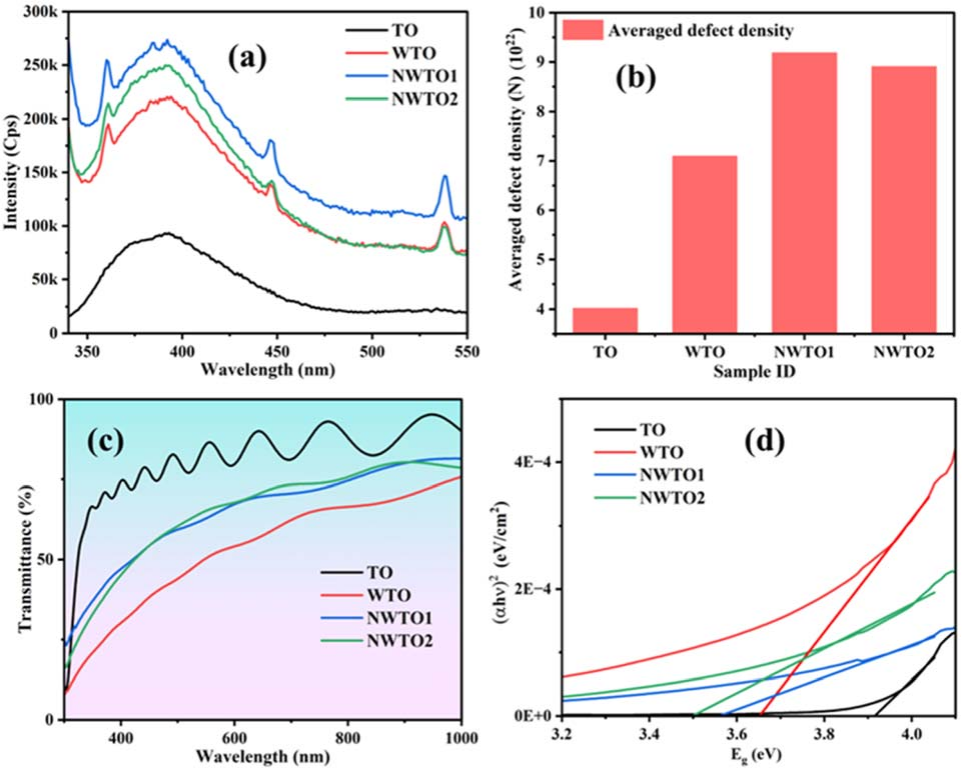
(a) Photoluminescence emission plots, (b) defect density, (c) transmittance curves, and (d) Tauc plot of the spray-pyrolyzed pure and Nb:W codoped SnO_2_ films.

The photoluminescence (PL) emission spectra of pure and Nb:W co-doped SnO_2_ films were recorded at room temperature using an excitation wavelength of 325 nm. PL emission curves for the Nb:W co-doped and pure SnO_2_ films assessed from 340 to 550 nm are displayed in Fig. 5a. The deposited SnO_2_ film shows a broad emission curve termed violet emission in the range of 360–430 nm centred at 390 nm. This is also referred to as near-band edge emission and represents the flow of electrons from the valence band maxima to the conduction band minima. The incorporation of Nb and W into the SnO_2_ lattice has enhanced the PL intensity thereby creating new defect levels and impurity states in the semiconductor lattice. The increased luminescence has emerged as a consequence of the transition of charge carriers between the defect energy levels acting as recombination foci for excitons. The defect-mediated luminescence emission intensity is significantly influenced by the density of oxygen vacancies.^30^ Apart from the UV emission peak, two peaks in the visible region were identified at about 440 and 540 nm, which correlate with blue and green emission. The luminescence sites created by Sn interstitials (Sn_i_) or dangling bonds observed in the SnO_2_ thin films are responsible for the base of the curve at 450 nm^31,32^ (2.75 eV). Point defects, including oxygen vacancies, tin interstitials, and metallic tin, are known to affect visible emission. Smakula's equation (eqn (5)) is utilized to estimate the collective defect density *N* of the spray-pyrolyzed pure and Nb:W co-doped SnO_2_ thin films.^33^5
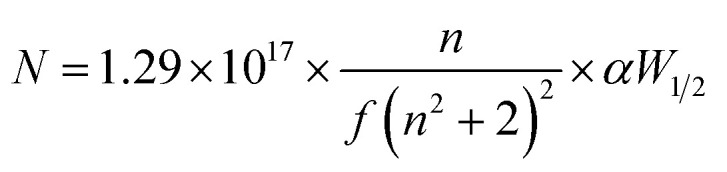


The area of the Gaussian curve is represented by *αW*_1/2_, *n* is the index of refraction of SnO_2_ (*n* = 2), and *f* (=1) is the strength of the oscillator. The calculated defect density values for all doped samples are higher than that of the pure sample due to the increase in oxygen vacancies brought about by doping (Fig. 5b). This additionally clarifies the significant reduction in crystallite size growth and alteration of surface morphology of the solely W and Nb:W co-doped SnO_2_ films from XRD and FESEM analyses, respectively.

The UV-Vis transmittance spectra of pure and Nb:W co-doped SnO_2_ thin films measured at ambient temperature in the range 300 nm to 1000 nm are shown in Fig. 5c. The primary variables that influence a thin film's transmittance are its microstructure, composition of dopants, thickness of the film, surface roughness, and coating parameters. Defect density (Fig. 5b) from PL shows that the introduction of defects and impurity states, which alter the material's optical characteristics, is principally responsible for the drop in transmittance seen after doping the SnO_2_ films. The presence of defects beneath the conduction band acts as localized energy states to trap charge carriers, resulting in enhanced absorption of light of a specific wavelength. Consequently, more light is absorbed than is transmitted through the film, leading to lower total transmittance.

The Tauc plot, displaying the correlation between the absorption coefficient (*α*) and incident photon energy (*hυ*), can be applied for assessing the optical energy band gap of the coated SnO_2_ films. As indicated in Fig. 5d, the band gap can be obtained by extrapolating the linear fit region of the plot to energy in the *X*-axis. The absorption coefficient (*α*) is calculated using eqn (6):6
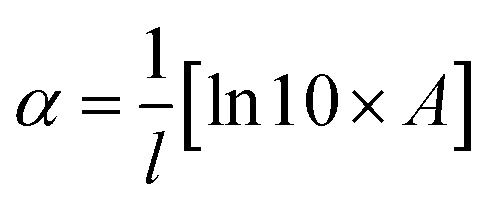
where *A* and *l* are termed absorbance and film thickness, respectively.

The bandgap of the deposited films has considerably reduced after the incorporation of dopants, implying alteration in the electronic states. The reduction in transmittance is a clear indication of inclusion of additional electronic states, which promotes the narrowing of the bandgap. Subsequently, following interactions between electrons and impurities, the electronic states of the SnO_2_ lattice undergo modifications at high concentrations of carriers beyond the Mott critical concentration, or >10^20^ cm,^3,34,35^ causing the band gap to decrease.

### Chemical charge state analysis

**Fig. 6:**
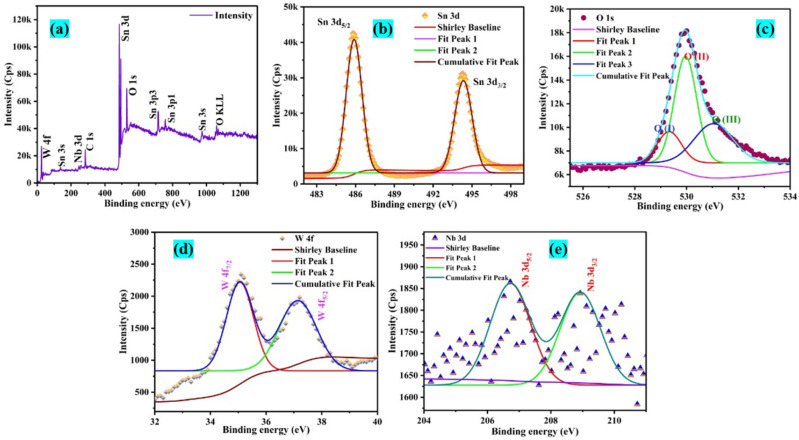
(a) Full survey spectrum and narrow scan peaks of (b) Sn 3d, (c) O 1s, (d) W 4f, and (e) Nb 3d elements present in the NWTO2 thin film.

X-ray photoelectron spectroscopy (XPS) analysis is used to determine the binding energies of the elemental constituents of the optimal sample NWTO2. The resulting plots are shown in Fig. 6, along with details of the charge states of the constituent elements. The full survey scan of the spray coated SnO_2_ films is presented in Fig. 6a along with individual core-level scans of Sn 3d (Fig. 6b), O 1s (Fig. 6c), W 4f (Fig. 6d), and Nb 3d (Fig. 6e). The existence of Sn, W, Nb, and O elements in the respective charge states in each film is confirmed using the XPS spectra. The charge states of the W and Nb elements determined using the binding energy value support its integration at the Sn sites. The charge state of Sn^4+^ is indicated by binding energies at 485.9 and 494.3 eV in the core-level spectra of the Sn 3d_5/2_ and Sn 3d_3/2_ oxidation states of all three samples. The peak positions shown ensure the formation of Sn–O and Sn–Nb bonds in the SnO_2_ matrix.^36^ The asymmetric O 1s core level spectrum has now been deconvoluted into three parts; the O(i) peak indicates that oxygen is bonded to Sn; the O(ii) peak indicates that Sn is present at the nearest neighbour oxygen vacancy (V_O_); the O(iii) peak indicates that adsorbed H_2_O, chemisorbed surface hydroxyl, and/or CO_2_ are present.^37^ The spin–orbit splitting of W 4f states is witnessed at binding energy values of 35.04 and 37.19 eV for W 4f_7/2_ and W 4f_5/2_, respectively. These results indicate that tungsten is oxidized to the 6+ valence state and the majority of W^6+^ is incorporated into the SnO_2_ matrix at Sn sites. Furthermore, the core-level scan of Nb 3d presents two symmetric peaks at 206.7 and 208.9 eV that are attributed to Nb 3d_5/2_ and Nb 3d_3/2_ doublets, respectively, and can be ascribed to Nb^5+^.

### Electrical properties

The electrical transport characteristics of the spray-deposited pure and Nb:W co-doped SnO_2_ films were investigated at room temperature using the Hall effect and four probe methods. All the films have been identified to have n-type conductivity based on the data obtained from the Hall effect characterization. The electrical characteristics of the spray-deposited films, such as mobility (*μ*), resistivity (*ρ*), sheet resistance (*R*_s_), and carrier concentration (*n*), are compiled in Table 3. Introduction of defect levels in the band gap of the doped samples has led to the donation of free electrons (n-type doping), which increases the material's total charge carrier concentration.^38^ In n-type doping, dopants replace the host atoms in the lattice structure, contributing additional free electrons that aid in the conduction process. The increase in the carrier concentration can enhance the conductivity of the sample, thus making it more beneficial. The decrease in the resistivity value from pure to doped samples can be understood easily from the aforementioned concept. The smaller particle formation seen in FESEM images can result in a higher density of grain boundaries, which further act as potential scattering sites for charge carriers thereby impeding their mobility.^39^ The reduction in particle size and increase in the carrier concentration contribute towards the lowering of the mobility value for the doped samples by minimizing the effective transport pathways. The sheet resistance decreased significantly after the incorporation of dopants in the deposited SnO_2_ thin films. An increased concentration of charge carriers promotes current conduction, hence reducing the resistance produced by the material when electric current flows through it. The increase in sheet resistance of NWTO2 may be due to the higher level of doping which raises the scattering of the additional charge carriers which eventually diminishes the mean free path thereby decreasing conductivity.

**Table 3:** Optical and electrical transport features of the spray-pyrolyzed pure and Nb:W co-doped SnO_2_ films

Sample ID	Band gap (eV)	Carrier concentration (*n*)	Resistivity (*ρ*)	Mobility (*μ*)	Sheet resistance Ω □^−1^	
					Hall effect	Four probe
TO	3.91	3.57 × 10^19^	1.16 × 10^−2^	15.04	132	118
WTO	3.65	3.88 × 10^20^	3.21 × 10^−3^	6.11	62	65
NWTO1	3.56	2.43 × 10^20^	4.29 × 10^−3^	6.28	83	75
NWTO2	3.50	1.82 × 10^20^	8.50 × 10^−3^	6.49	173	72

**Fig. 7:**
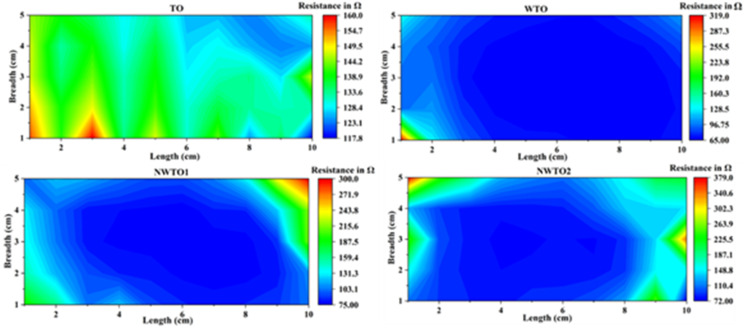
2D contour mapping of sheet resistance over a 10 × 5 cm^2^ area of pure and Nb:W co-doped spray deposited SnO_2_ thin films.

The outcomes of the 2D contour map plot of sheet resistance for all the coated films, assessed over a 10 × 5 cm^2^ area (Fig. 7), indicate that the resistance values are low and rather uniform in the middle, with a slight divergence towards the periphery. The insertion of dopants has enhanced the sheet resistance of the co-doped SnO_2_ thin films. The main reason for the variation of the *R*_s_ value between the four-probe and Hall effect techniques is the fundamentally varying approaches used in these methods.^40,41^ Furthermore, the data obtained from the four-probe measurement indicate that the Rs measured cover the entire film surface, while the Hall effect is observed only over a 1 cm^2^ area. Eqn (7) can be used to compute the sheet resistance *R*_s_ by evaluating the potential difference between the pair of middle probes (*V*) and applied current (*I*) between the pair of outer probes.7
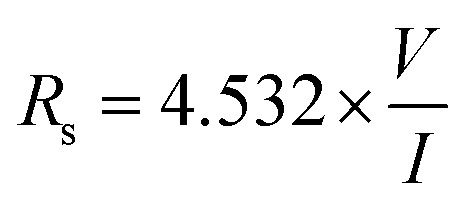


A correction factor of 4.532 is included depending on the probe configuration since the thickness of the film is relatively smaller compared to the probe spacing (about 1 mm); *V* and *I* represent voltage and applied current, respectively. The singly W doped SnO_2_ film has a minimal sheet resistance of 65 Ω □^−1^, which is also consistent with the Hall effect findings.

### Photocatalytic activity measurements

**Fig. 8:**
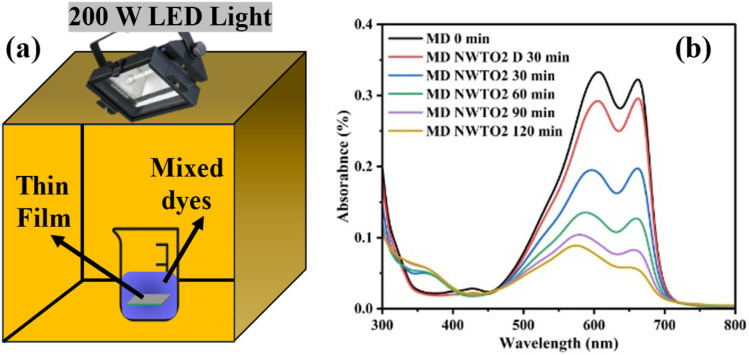
(a) Schematics of the photocatalytic experimental arrangement and (b) absorbance spectra of the degraded MD dye for the spray deposited NWTO2 photoelectrode.

The potential of multi-component dye solution photocatalytic activity to achieve pollutant degradation with low energy consumption has drawn attention in recent studies.^42,43^ NWTO2 is chosen as a suitable sample for photocatalytic studies based on its lower band gap, optimum sheet resistance and comparatively high transmittance; photolysis and the TO film are utilized for comparison. To illustrate the dye degradation in visible LED light, the mixed solution of organic pollutants was selected to serve as a model pollutant. The experimental setup with a 200 W LED lamp is presented in Fig. 8a, which is being used to conduct the photocatalytic process as detailed in Section 2.3.

**Fig. 9:**
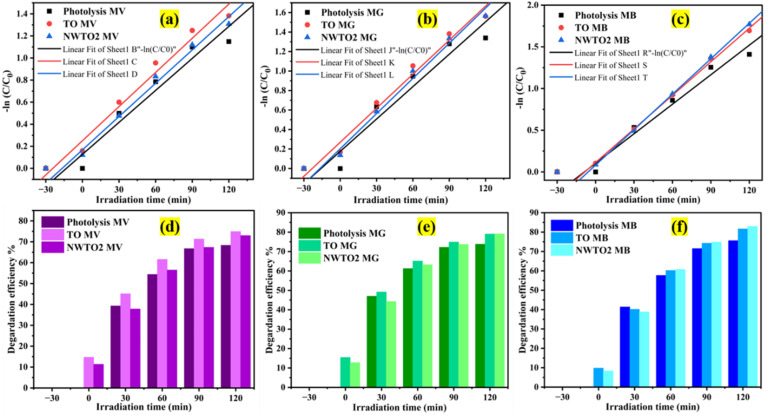
(a) 1st order rate constants and disintegration efficiency of mixed dye components (a and d) MV, (b and e) MG, and (c and f) MB as an outcome of degradation with different irradiation times, respectively.

Fig. 8b displays the UV-Vis absorption spectra of the mixed dyes acquired through photodegradation using a 200W LED light-illuminated NWTO2 electrode and recorded every 30 minutes. The integration of several dye structures results in the development of many absorption peaks in the spectra. In addition, the absorbance rapidly decreased in the first half hour of irradiation, decolorizing the mixed dyes to almost half of its initial value. The absorbance values dropped slower upon subsequent radiation, potentially as there were considerably fewer probabilities that the dye molecules would be destroyed by oxidizing agents and/or by the adsorbed holes at low concentrations. Dark adsorption is done for 30 minutes to reach saturation (D 30 min) before the photocatalytic activity is measured. The kinetics in the presence of both pure and Nb:W doped SnO_2_ films were evaluated so as to obtain a more accurate and quantitative understanding of photodegradation of the dye. The plot of −ln(*C*/*C*_0_) *vs.* irradiation time illustrates the pseudo-first-order kinetics of the MD solution photodegradation by the films (Fig. 9a–c). The calculated slope (also known as rate constant *k*) value for the curves of individual dyes is found to be highest for the sample NWTO2. The fact that the dopants W and Nb contributed considerably to determining the rate of photodegradation is indicated by their rate constant (*k*) values being larger than those of TO and photolysis.

The disintegration efficiency (*η*) of photolysis and photocatalysis was assessed using the initial and final concentrations of the MD solutions, as illustrated in Fig. 9d–f. The photocatalytic efficiency is found to be higher in the presence of the photoelectrode indicating the active participation of charge carriers generated from the electrode surface upon light illumination. The degradation efficiency of the individual dye components in the mixture is estimated from the peaks at respective absorption maxima. The NWTO2 film showed a better efficiency for MG and MB dyes, while in the case of MV dye, the TO film showed a better efficiency. It is noteworthy that a remarkable efficacy is reached without any turbulence in the photocatalytic setup. A semiconducting photocatalyst's structure, phase, morphology, film thickness, and film stability are some of the significant factors influencing its activity during the degradation process. Mohan *et al.* discovered that the reaction rate of the photocatalytic and stability are significantly influenced by the catalyst's film thickness.^44^ The high efficiency of the TO film can be attributed to better transmittance and high mobility, which enhanced the light absorption over the complete photocatalyst layer thickness and low recombination rate of charge carriers, respectively, that permit charge diffusion and utilization at the redox sites.

**Fig. 10:**
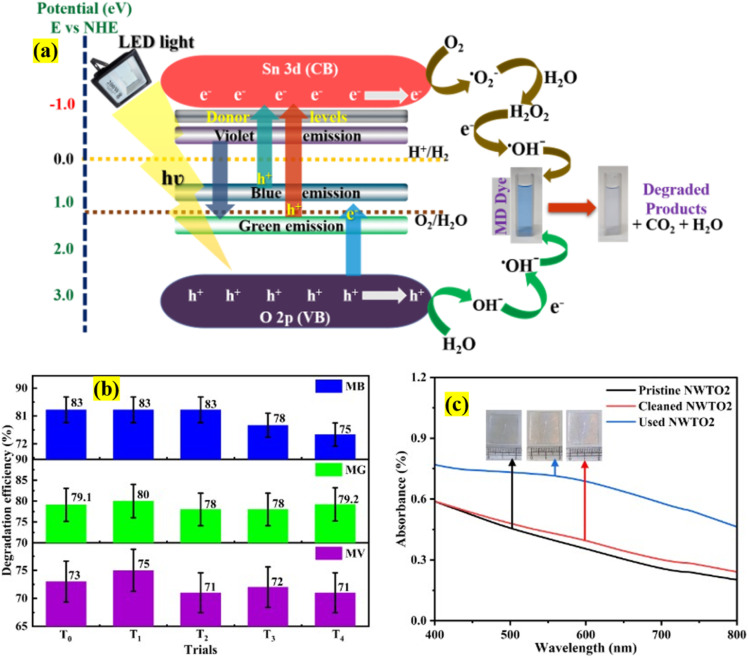
(a) Plausible charge transfer mechanism for the MD dye degradation, (b) recyclability test showing variation in disintegration efficiency from the pristine (T_0_) attempt to four re-use trials (T_1_ to T_4_) and (c) absorbance spectrum of the as-prepared, used, and cleaned surface following the photocatalytic reaction with the NWTO2 catalyst. The standard error is also denoted.

The primary goal of this work is to study transitions in defect levels existing within the bandgap of the material under exploration, despite the fact that the sample's band edge is in the ultraviolet range. The presence of point defects such as oxygen vacancies is already confirmed from the blue and green emission of the PL data (Fig. 5a). The degradation mechanism initiates when the white light is irradiated on the semiconductor catalyst surface generating electron–hole pairs (Fig. 10a). Since the light energy (*hʋ* < *E*_g_) is insufficient to transfer the electrons from the valence band of O 2p to the conduction band of Sn 3d, charge carriers are separated through transitions from band levels to defect levels. As described in the PL section, the three peaks of violet, blue, and green emissions indicate the presence of defect levels predominantly within the band gap at 3.17 eV (390 nm), 2.81 eV (440 nm), and 2.25 eV (550 nm), respectively. Upon irradiation of light the charge separation process takes place by the transitions between these defects and band levels. Furthermore, defect states operate as a trap, preventing carrier recombination and thereby extending the life of charge carriers. Additionally, the donor impurities of Nb and W introduce new energy levels within the band gap that are close to the conduction band. These donor levels enable electrons to be easily triggered into the conduction band, increasing the quantity of free electrons and improving the semiconductor's conductivity. The excited electron leaves a hole in the valence band by jumping to the defect states that lie below the conduction band. The dissolved oxygen (O_2_) and water molecules (H_2_O) in the mixture react with these charge-separated electron–hole pairs, respectively, resulting in reduction-oxidation processes that create superoxide and hydroxyl radicals, which further contribute to the MD solution's deterioration.^45^

Nevertheless, the valence band position for film NWTO2 was estimated using the Butler–Ginley equation.^46^ The mathematical equations given below are used for determining the NWTO2 thin film's valence band potential using absolute electronegativity.

8 *E*_VB_ = *χ* − *E*_e_ + 0.5*E*_g_

9 *E*_CB_ = *E*_g_ − *E*_VB_

The semiconductor's equation comprises electronegativity (*χ*), band gap energy (*E*_g_), and free electron energy (*E*_e_), with a typical value of 4.5 eV *vs.* NHE. The *E*_VB_ and *E*_CB_ positions have been determined to be 2.58 eV and −0.92 eV, respectively. The above data ensure that the VBM and CBM positions fulfil the half-reaction potentials H^+^/H_2_ and O_2_/H_2_O (water splitting levels), respectively. Considering the above data with respect to the photocatalytic degradation of MD dye in the presence of the NWTO2 catalyst the following probable mechanism (Fig. 10a) and chemical reactions are discussed which are also applicable to the pure SnO_2_ thin films,

10 Nb:W:SnO_2_ + *hν* → e_CB_^−^ + h_VB_^+^

11 h_VB_^+^ + H_2_O → OH^.^+ H^+^

12 e_CB_^−^ + O_2_ → ˙O_2_^−^

13
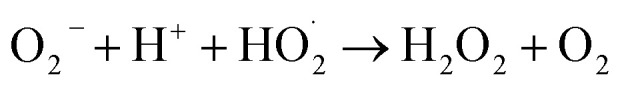

14 H_2_O_2_ + *hν* → 2HO

15 MV dye + *hν* → MV dye*

16 OH˙/O_2_˙^−^ + MV dye/MV dye˙^+^ → Degraded Products

Table 4 shows the photocatalytic results obtained for the MD solution degradation with photolysis for the optimum NWTO2 and pure TO films. The spray-deposited films exhibited significant textured growth along the (110) and (200) plane directions, contributing to increased photocatalytic performance.^47^ Despite the fact that pure SnO_2_ has a high textured growth, the addition of Nb and W has greatly enhanced the porosity of the film, which improves the surface contact area of the photocatalyst with the pollutant. Furthermore, oxygen vacancies (V_o_), identified by PL analysis (Fig. 5a and b), provide localised mid-gap states that operate as electron traps, lowering recombination rates and prolonging charge carrier lifetimes.^48^ The relatively higher content of the O(ii) peak (Fig. 6c) reveals the V_o_-rich nanostructured NWTO2 thin film, which serves as a better O_2_ activator due to improved adsorption and charge transfer kinetics.^49^ These vacancies enhance the accessibility of active sites for surface reactions, allowing V_o_ to directly engage in redox processes, such as generating hydroxyl radicals (˙OH) during pollutant breakdown.^50^ Sn-based defects, especially interstitials and dopants, alter photocatalytic activity *via* band structure modification and charge carrier dynamics. Sn interstitials (Sn_i_) are cationic defects in which additional Sn atoms occupy interstitial locations in the SnO_2_ lattice. Compared to oxygen vacancies, Sn_i_ demonstrate remarkable stability in varied chemical circumstances due to Sn's multivalent nature.^51^ Sn_i_ contribute energy levels in the band structure of SnO_2_, forming mid-gap states or shallow donor levels. These defect states influence the charge carrier capturing and recombination mechanisms required for photocatalysis. In particular, Sn_i_ can operate as electron traps, altering electron–hole recombination rates by delaying or providing alternate recombination paths.^52^ Collectively, Sn interstitials and oxygen vacancies shrink the bandgap for visible-light absorption, enhancing photocatalytic efficiency. The suitable defect density and carrier concentration of the NWTO2 film cooperate synergistically to minimize the impact of charge carrier recombination effects.

**Table 4:** 1st order rate constants and disintegration efficiency of spray-deposited SnO_2_ thin films along with photolysis for the MD solution under white LED light

Dye component	Rate constant (*k*)			Disintegration efficiency (*η*) %		
	Photolysis	TO	NWTO2	Photolysis	TO	NWTO2
MV	0.009	0.010	0.010	69	75	73
MG	0.010	0.011	0.012	73	79	79.1
MB	0.011	0.013	0.014	75	81	83

Cyclic studies are essential for analysing the prolonged reliability, performance, and applicability of photocatalysts, all of which are required for their effective usage in practical applications.^53^ The practicality of the NWTO2 thin film electrode's photocatalytic activity in degrading the MD dye was assessed by conducting four consecutive re-use cycles along with the pristine trial on the same electrode piece. Following every re-use experiment, the thin-film electrode was thoroughly cleaned with deionised water, ultrasonicated using isopropyl alcohol for ten minutes, and dried to restore the electrode surface by eliminating the adsorbents. The recyclability test of the NWTO2 film was performed under nearly identical experimental conditions, with the results presented in Fig. 10b. The cyclic photocatalytic measurement in the presence of visible light shows that the rate of MD dye degradation efficiency diminishes marginally with each consecutive use of the NWTO2 photocatalyst thin film catalyst. There are certainly many possibilities for the alteration in disintegration efficiency from first to fifth re-use trials; repetitive exposure to the light and reacting agents may stimulate or clean the photocatalyst surface, increasing active sites and efficiency.^54^ During rejuvenation treatments (such as surface cleaning by washing), the removal of adsorbed contaminants or intermediates (Fig. 10c: absorption spectra of cleaned NWTO2) restores and revives catalytic activity.^55^ Also, adsorption of organic or inorganic impurities (Fig. 10c: absorption spectra of used NWTO2) can change the surface chemistry, preventing charge transfer or reactive species production. Eventually, the degradation efficiency declines with repeated usage (up to ∼8% for MB dye, 1-2% for MG dye, and 2-4% for MV dye) due to factors such as an accumulation of residual organic pollutants on the catalyst electrode's surface and/or pores, leaching of film from multiple uses, and fluctuations in light intensity from excessive usage with time. However, the drop in performance upon re-use cycles is relatively insignificant, showing that the film's adhesion and photocatalytic activity are quite durable. The effectiveness of Nb:W co-doped SnO_2_ films against other TCOs as a photocatalyst was compared to other reported metal oxide thin films (Table 5). As demonstrated in Table 5, the deposited film has an unambiguous degrading potential when compared to other published TCO based catalysts provided the parameters, choice of dye, pollutant volume, catalyst area and light source are taken into account. Based on the findings presented in Table 5, it is apparent that the present work uses cost-effective LED light to efficiently degrade the multicomponent solution within 120 minutes. In contrast the reported studies mostly rely on the use of UV (harmful) and high wattage lamps (power consuming) for the deterioration of a single dye solution. The cyclic test of the Nb:W co-doped SnO_2_ thin film photocatalyst demonstrates that the electrodes are stable enough for continuous usage with only a low reduction in photocatalytic efficiency. The reasons stated above demonstrate the genuine efficacy of the deposited films against the reported TCO-based photocatalysts. Hence, despite the Nb:W codoped SnO_2_ photocatalyst electrode's extremely thin dimensions (in relation to photocatalysts in powder form), it appears to be a system that is sufficiently stable to potentially lower the pollutant content in water, reducing the cost of treating dyes in contaminated water.

**Table 5:** Comparison of photocatalytic performance of Nb:W co-doped SnO_2_ electrodes *vs.* that of other TCO-based photoelectrodes

Sl. No.	Photoelectrode – parameters	aDye for degradation	Pollutant volume/catalyst area/light source	Performance highlights (resources)
1	TiO_2_ – varying calcination temperature	Degradation of MO	25 ml/80 cm^2^/15 W UV lamp	TiO_2_ film calcined at 700 °C showed the highest rate constant of 0.0018 min^−1^ (ref. 8)
2	TiO_2_ – Sn ion implantation	Degradation of Rh B	5 ml/5 cm^2^/8 W UV lamp	Rh B was degraded up to 80% after 160 min (ref. 9)
3	TiO_2_ – thickness variation	Degradation of SA	100 μL/4 cm^2^/UV-A lamp	TiO_2_ film of thickness 190 nm showed the highest activity with a rate constant 0.01648 min^−1^ (ref. 10)
4	Al/TiO_2_ – thermal deposition of the Al layer	Rh B	1.5 cm^2^/sunlight	An abatement of 30% was obtained for the degradation of Rh B^11^
5	TiO_2_–ZnO – Ag doping	Degradation of MB	2 ml/1 cm^2^/15 W UV lamp	2 mol% Ag-doped TiO_2_ – ZnO films showed an efficiency of 80% with a rate constant of 0.758 h^−1^ (ref. 12)
6	ZnO – Cu doping	Degradation of orange II	Visible light	Pure ZnO film showed 72% degradation efficiency and it decreased with increasing Cu doping^13^
7	ZnO – multilayer film (ZnO/AZO/ZnO) with Al doping	Degradation of MB	40 ml/24 cm^2^/750 W	Maximum efficiency of 95.2% with a rate of 1.02 h^−1^ was obtained at 20% Al doping^14^
8	ZnO – Al doping	Degradation of MB	50 ml/1 cm^2^/15 W UV lamp	Al doped ZnO films showed an efficiency of 80% with a rate constant of 0.002 min^−1^ (ref. 15)
9	ZnO – Co doping	Degradation of MB	50 ml/1 cm^2^/100 W	15 wt% Co doped ZnO film showed 100% degradation with a rate constant of 0.013 min^−1^ (ref. 16)
10	SnO_2_ – Fe, Ni doping	Degradation of MB	15 ml/11 W UV lamp	∼85% efficiency was obtained for the Fe and Ni doped SnO_2_ with a rate constant of 0.004 min^−1^ (ref. 17)
11	ZnO and SnO_2_ – substrate modification (polytherimide and glass)	Degradation of CV	50 ml/2 cm^2^/100 W UV lamp	ZnO and SnO_2_ films (with a polytherimide substrate) showed an efficiency of 80 and 85% with a rate constant of 0.009 and 0.006 min^−1^ (ref. 18)
12	SnO_2_ – F doping	Degradation of CV	50 ml/2 cm^2^/100 W UV-lamp	Degradation efficiency increases to 96% and 92% for pure and F: SnO_2_ on adding H_2_O_2_ respectively^19^
13	SnO_2_ – Ce doping	Degradation of MB	20 ml/3.75 cm^2^/UV lamp	Degradation of 19.10% is obtained for 2 wt% Ce:SnO_2_ (ref. 20)
14	SnO_2_ – Sr doping	Degradation of MB	20 ml/15 W UV lamp	Degradation efficiency of 38% was achieved for an 8 wt% Sr doped SnO_2_ film with a rate constant of 0.005 min^−1^ (ref. 21)
15	SnO_2_ – Nb, W doping	Degradation of (MV + MG + MB) MD	6 ml/4 cm^2^/200 W LED light	Significant degradation efficiency (73%:methyl violet, 79.1%:malachite green, and 83%:methylene blue) is achieved within 120 min (present work)

a Methylene Blue – MB, Rhodamine B – Rh B, Methyl Orange – MO, Stearic Acid – SA, and Crystal Violet – CV.

## Conclusions

Pure and Nb:W co-doped SnO_2_ films with a tetragonal SnO_2_ phase devoid of additional peaks associated with secondary phases were deposited using the facile spray pyrolysis procedure. The compact and uniform surface morphology transforms into a unique network-like structure after the addition of W delivering the possibility for tailoring the morphology to suit specific needs. The growth kinetics have been dynamically altered by Nb and W doping, yielding a relatively smoother surface as the contact angle increases in relation to increasing doping content. The optical transmittance of pure SnO_2_ films indicates a high transparency of 86% with a wider band gap of 3.91 eV while the co-doping with Nb:W has narrowed down the bandgap to 3.50 eV for NWTO2 enabling visible light absorption while reducing the transmittance to 66%. The development of newer impurity levels within the optical band gap is the primary reason for the reduction in bandgap. An increase in the carrier concentration from pure to Nb:W co-doped SnO_2_ has enhanced the electrical conductivity while suppressing the mobility of charge carriers by limiting the mean free path. Consequently, the inclusion of W as a dopant enhances the sheet resistance's general uniformity; the WTO film's minimum value is estimated to be around 62 Ω/□. The boost in the photocatalytic process of NWTO2 for the deterioration of mixed dyes is a result of its ideal optoelectronic properties, which include the optimal defect concentration, transmittance, and a relatively smaller band gap which have contributed to separating the charge carriers and distributing visible light evenly across the film. The photoelectrodes are found to be cost-effective and durable catalysts for the degradation of dyes, with the capacity to degrade multiple dyes in the range of 73 to 83% simultaneously under 200 W LED light without requiring assistance of turbulence in the system. The cyclic test performed under near identical conditions revealed that the photoelectrodes are stable enough up to five cycles indicating their practical applicability. In addition, in contrast to powder and particulate films, thin films in photocatalysis possess numerous advantages, including better surface area interaction, minimal secondary contamination, higher light absorption, easy cleaning, improved durability, and simple device integration.

## Data availability

The additional data related to this work are given in the ESI† and the original raw data pertaining to the work presented in this article will be shared based on the necessity and request.

## Author contributions

Conceptualization, D. P. J. and G. M.; investigation, G. M. and D. P. J.; methodology, D. P. J. and G. M.; resources, D. P. J. and M. K.; supervision, D. P. J.; writing—original draft, G. M.; writing—review and editing, D. P. J., G. M. and Y. J.

## Conflicts of interest

There are no conflicts to declare.

## Supplementary Material

NA-OLF-D5NA00268K-s001
