# Supplementary material for: Photodegradation of mixed organic dyes using Nb and W co-doped spray coated transparent conducting SnO2 thin films
Source: Nanoscale Adv. 2025 Jun 3;7(13):4169–82. doi: 10.1039/d5na00268k (PMC12131908; doi:10.1039/d5na00268k)
Supplement: NA-007-D5NA00268K-s001 [file NA-007-D5NA00268K-s001.pdf]

Supplementary Information (SI) for Nanoscale Advances.
This journal is © The Royal Society of Chemistry 2025

Photodegradation of Mixed Organic Dyes using Nb and W Co-doped Spray Coated Transparent Conducting SnO_2 Thin Films

Gouranga Maharana[a], Yuvashree Jayavelu[a], Manavalan Kovendhan[b], D. Paul Joseph[a*]

[a]Department of Physics, National Institute of Technology, Warangal, Telangana State-506004, India.
[b]Department of Physics and Nanotechnology, SRM Institute of Science and Technology, Kattankulathur, Tamilnadu-603203, India.
*Corresponding author e-mail: paul@nitw.ac.in

S1. The visible light spectrum of 200 W LED

To achieve photocatalytic activity through defect mediated mechanism of SnO_2 thin films, the white visible light is used for the test. The figure below represents the light spectrum of the used 200 W LED light in order to understand the light energy received by the SnO_2 photoelectrodes to carry out the photocatalytic tests. This spectrum clearly shows that the white light emitted by the LED consists of visible region wavelength with two major peaks around 450 and 530 nm respectively.

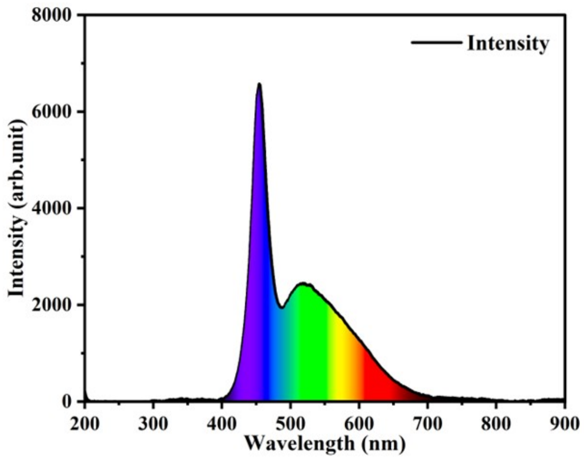

Fig. S1. The visible light spectrum of the 200 W LED light used for the photocatalytic study showing sharp peak intensity around 450 nm and a broad peak at 530 nm.

****
